# Re-Directing an Alkylating Agent to Mitochondria Alters Drug Target and Cell Death Mechanism

**DOI:** 10.1371/journal.pone.0060253

**Published:** 2013-04-09

**Authors:** Rida Mourtada, Sonali B. Fonseca, Simon P. Wisnovsky, Mark P. Pereira, Xiaoming Wang, Rose Hurren, Jeremy Parfitt, Lesley Larsen, Robin A. J. Smith, Michael P. Murphy, Aaron D. Schimmer, Shana O. Kelley

**Affiliations:** 1 Department of Pharmaceutical Sciences, Faculty of Pharmacy, University of Toronto, Toronto, Ontario, Canada; 2 Department of Biochemistry, Faculty of Medicine, University of Toronto, Toronto, Ontario, Canada; 3 Princess Margaret Hospital, Ontario Cancer Institute, Campbell Family Cancer Research Institute, Toronto, Ontario, Canada; 4 Department of Pathology, University of Western Ontario, London, Ontario, Canada; 5 Department of Chemistry, University of Otago, Dunedin, New Zealand; 6 Medical Research Council Mitochondrial Biology Unit, Wellcome Trust/Medical Research Council Building, Cambridge, United Kingdom; Univ of Bradford, United Kingdom

## Abstract

We have successfully delivered a reactive alkylating agent, chlorambucil (Cbl), to the mitochondria of mammalian cells. Here, we characterize the mechanism of cell death for mitochondria-targeted chlorambucil (mt-Cbl) *in vitro* and assess its efficacy in a xenograft mouse model of leukemia. Using a ρ° cell model, we show that mt-Cbl toxicity is not dependent on mitochondrial DNA damage. We also illustrate that re-targeting Cbl to mitochondria results in a shift in the cell death mechanism from apoptosis to necrosis, and that this behavior is a general feature of mitochondria-targeted Cbl. Despite the change in cell death mechanisms, we show that mt-Cbl is still effective *in vivo* and has an improved pharmacokinetic profile compared to the parent drug. These findings illustrate that mitochondrial rerouting changes the site of action of Cbl and also alters the cell death mechanism drastically without compromising *in vivo* efficacy. Thus, mitochondrial delivery allows the exploitation of Cbl as a promiscuous mitochondrial protein inhibitor with promising therapeutic potential.

## Introduction

The nitrogen mustard chlorambucil (Cbl) was one of the first anti-cancer drugs to be developed and used clinically [1], [2]. This DNA alkylating agent was engineered from an original set of compounds, which included drugs such as chlormethine, to have a more favorable kinetic profile and decreased toxicity [3], . Chlorambucil functions as a mono- or di-alkylating agent by reacting primarily with the N7 of guanine to produce intra- or inter-strand crosslinks [5]. Formation of these crosslinks often stalls replication and transcription, leading to cell cycle arrest and apoptosis [6], [7]. Cbl has successfully been used to treat leukemia and while the initial response rate in patients is typically 40–60%, complete remission is rare [8]. Instead, patients often develop resistance to the drug necessitating the use of other agents, such as fludarabine [9], [10].

**Figure 1:**
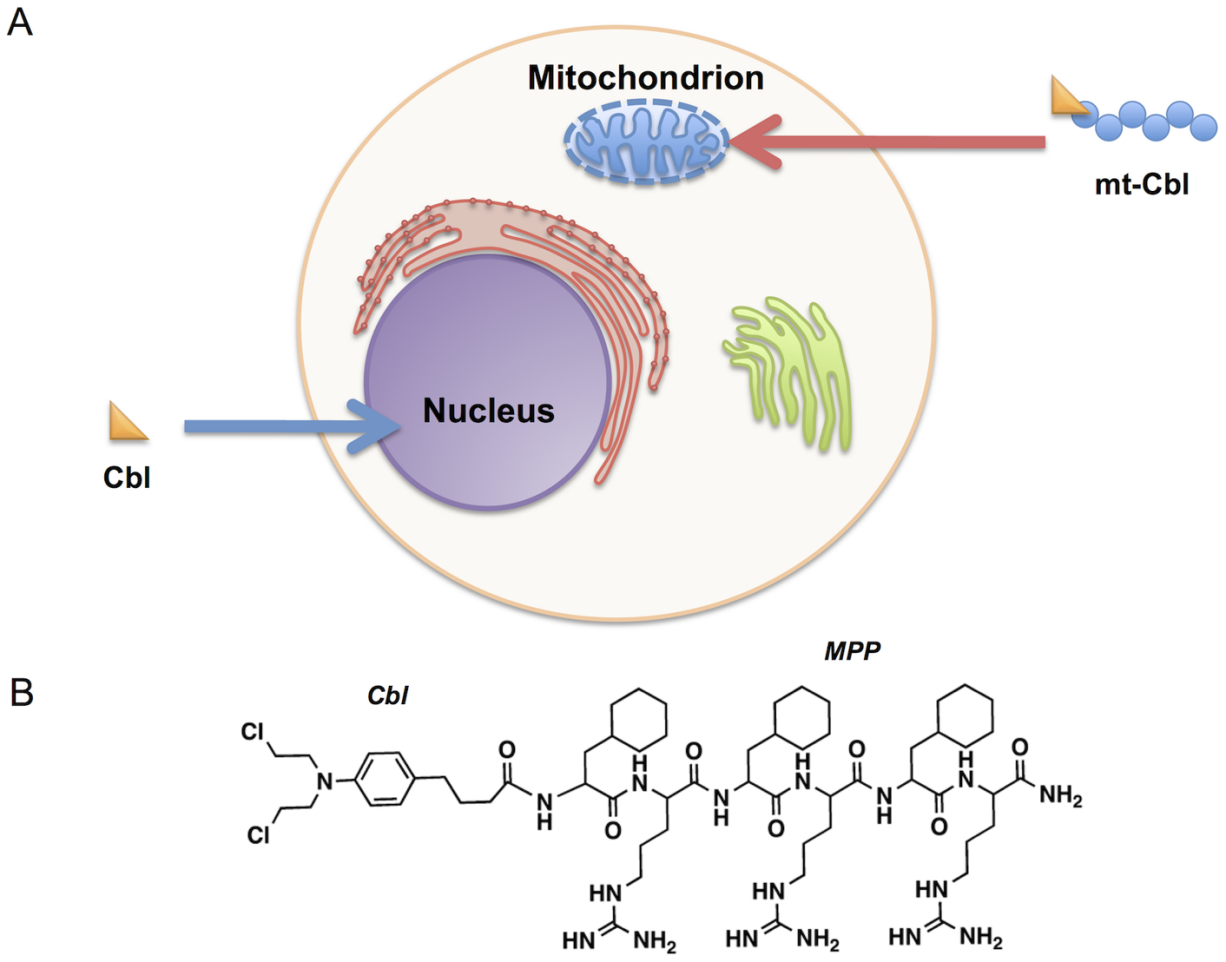
Mitochondrial rerouting of chlorambucil. (a) ***Schematic of chlorambucil redirected to mitochondria.*** Conjugation of chlorambucil (Cbl) to a mitochondria-penetrating peptide (MPP) sequence (mt-Cbl) redirects Cbl from its usual nuclear target to the mitochondrion. (b) ***Chemical structure of mt-Cbl.*** Cbl is conjugated to a MPP through the N-terminus.

We hypothesized that drug retargeting to a novel cellular location could give the drug access to new targets. In previous work, we redirected Cbl from its primary site of action – the nucleus – to an alternate location, the mitochondrion, using a novel peptide-based delivery vector (Figure 1) [11]–[13]. The mitochondrion is the only organelle besides the nucleus containing DNA (mtDNA) and is centrally involved in programmed cell death execution. Using a peptide delivery vector, the Cbl compound was delivered specifically to mitochondria with a high percentage localized within the mitochondrial matrix as previously determined by fluoresence microscopy and transmission electron microscopy of dye and biotin labelled compounds, respectively [11]. We hypothesized that sending the drug to a new organelle might influence the effectiveness of Cbl especially in resistant cells, and we were able to show this effect in a series of *in vitro* studies that assessed a panel of cell lines with varying resistance to Cbl. The therapeutic window for mt-Cbl was also assessed in isolated patient cells, and was shown to be maintained for the mitochondria-directed drug.

Here, we investigate the *in vitro* mechanism of action and the *in vivo* characteristics of the first active alkylating agent retargeted to the mitochondria. We present the surprising finding that despite the ability of mt-Cbl to alkylate mtDNA, damage to mitochondrial DNA is not necessarily a dominant factor in mt-Cbl toxicity. In addition, the activity of mt-Cbl was found to occur though the activation of necrosis. Two different forms of mitochondria-targeted Cbl are studied, and both have the same activity profile indicating that this is a general phenomenon for this drug when delivered to mitochondria. Assessing activity in a mouse cancer model, we also show that conjugation of a mitochondria-penetrating peptide (MPP) to Cbl improves the pharmacokinetic profile of the drug and is effective at killing tumor cells *in vivo*. Lastly, we demonstrate that despite the necrotic character of mt-Cbl, the drug is well tolerated and no drug-induced toxicity is observed within the therapeutic window.

## Materials and Methods

### Compound Synthesis and Characterization

#### Peptide synthesis

Peptide scaffolds were synthesized at a 50 μmol scale on Rink amide MBHA resin (0.56 mmol/g, 100–200 mesh) (NovaBiochem) using a Prelude automated peptide synthesizer (Protein Technologies, Inc). For couplings, Fmoc-protected D or L-amino acids (4 equiv., Advanced ChemTech, Fmoc =  9-fluorenylmethyloxycarbonyl), HBTU (4 equiv., Protein Technologies Inc., HBTU =  *O-*(benzotriazol-l-yl)-*N*,*N*,*N*',*N*'-tetramethyl-uronium hexafluorophosphate), and NMM (8 equiv., Protein Technologies, Inc., NMM =  *N-*methylmorpholine) were stirred in *N,N*-dimethyl formamide (DMF) for 1 hour. Arginine residues were added using double couplings. The Fmoc protecting group on the N termini was removed using piperidine (20% v/v) in DMF (2×15 min).

#### Synthesis and characterization of peptide conjugates

Thiazole orange (*to*) was synthesized as described previously [14] and coupled to peptides using HBTU (4 equiv.), and DIPEA (8 equiv.) in DMF for 3 hours. Cbl (Oakwood Products, Inc.) was coupled to peptides using HBTU (4 eq) and DIPEA (4 eq) in DMF. For biotin- or alkyne-labeled peptide conjugates, resin was coupled to Fmoc-Lys(Biotin)-OH (Anaspec, Inc.) or Fmoc-L-propargylglycine (Advanced ChemTech) using HBTU (4 equiv.), and DIPEA (8 equiv.) in DMF for 2 hours. The N terminus of unconjugated peptides was capped using acetic anhydride, pyridine, and dichloromethane (DCM) (1∶5∶10) (2×10 min). Peptides were deprotected and cleaved using trifluoroacetic acid:triisopropylsilane (TFA/TIPS, 95∶5 v/v) for 2 hours and precipitated and washed using cold ether. Peptides were purified using reverse-phase HPLC on a C18 column using MeCN/0.1% TFA and H_2_O/0.1% TFA as mobile phases. Peptide conjugate identity was then confirmed by electrospray ionization (ESI) mass spectroscopy. To reduce Cbl degradation, purified Cbl-peptide conjugates were immediately flash-frozen and lyophilized. Quantification of *to*-labeled peptides and Cbl-conjugated peptides was done using an extinction coefficient of 63,000 M^−1^ cm^−1^ at 500 nm and 15,200 M^−1^ cm^−1^ at 258 nm, respectively, in H_2_O. A bicinchoninic acid (BCA) assay (Thermo Scientific) was used to quantify unlabeled peptides. *For the in vivo studies* the mt-Cbl peptide was purchased from Anaspec (Freemont, CA) at >90% purity. All compounds were stored dry and stocks were prepared in DMSO as required. For *in vivo* studies, compounds were diluted in 0.9% saline prior to injection into animals.

#### Synthesis and purification of Cbl-TPP

A mixture of chlorambucil (100 mg, 0.33 mmol), dicyclohexylcarbodiimide (130 mg, 0.49 mmol) and pentafluorophenol (70 mg, 0.36 mmol) were dissolved in anhydrous dimethylformamide (1 ml) with sonication (1 minute), left at room temperature for 30 minutes, then triphenylphosphonium propylamine hydrobromide (170 mg, 0.36 mmol), and triethylamine (50 μL, 0.36 mmol) were added and the mixture left at room temperature for 24 hours. The resultant mixture was filtered to remove the DCC urea byproduct which was washed with dichloromethane. The combined filtrates were washed with water then shaken well with brine to exchange the anion. The organic phase was dried, then evaporated *in vacuo* first by rotary evaporation followed by high vacuum for several hours. The resultant off white gum was purified by column chromatography over silica gel eluting with 10% methanol in chloroform which gave the product as a white gum (129 mg, 57%). The identity of the product was confirmed by NMR and high resolution mass spectrometry.

### *In vitro* Studies

#### Cell culture

HeLa cells (ATCC) were cultured in Minimum Essential Medium alpha (Invitrogen) supplemented with 10% (v/v) fetal bovine serum (FBS, Sigma-Aldrich) on 75 cm^2^ cell culture plates with vent caps. HL60 cells (ATCC) and K562 cells (ATCC) were cultured in RPMI 1640 (Invitrogen) plus 10% FBS using suspension flasks with vent caps as described previously [11]. 143B cells (ATCC) were cultured in Dulbecco's Modified Eagle Medium with high glucose (DMEM, Invitrogen) plus 10% FBS. 143B ρ° cells were generously provided by Douglas Wallace (University of California, Irvine and originally described in [15]) and were cultured in DMEM high glucose plus 10% FBS supplemented with 100 mM sodium pyruvate and 5 mg/ml uridine. OCI-AML2 cells (established at the Ontario Cancer Institute [16]) were maintained in Iscove's modified Dulbecco's media supplemented with 10% FBS. All cell lines were incubated in a humidified incubator at 37°C with 5% CO_2_.

#### Cell viability assay

Adherent cell lines (HeLa, 143B and 143B ρ° cells) were seeded in 96-well flat-bottom tissue culture plates (Sarstedt) the day before treatment at a density of 12,000 cells per well. Cells were washed with serum-free media before treatment and pre-incubated with inhibitors at the indicated concentrations for 1 hour prior to the addition of chlorambucil or chlorambucil conjugates. The plates were incubated for 18 hrs at 37 °C in a humidified incubator and then 10 μL of CCK-8 viability dye (Dojindo, Rockville) was added to each well and the plates were further incubated till color development occurred. Absorbance at 450 nm was then measured and non-linear curve fitting and statistical analysis were performed using the GraphPad Prism software (GraphPad).

#### Annexin-V apoptosis assay

HeLa cells were seeded at 100,000 cells/well the day prior to an experiment in a 12-well flat-bottom tissue culture plate (BD Falcon). Chlorambucil and chlorambucil-conjugate at indicated concentrations were added to the cells in triplicate in serum-free cell-appropriate media. Cells were incubated for 90 minutes with mt-Cbl at 37°C with 5% CO_2_. Cells were later harvested and washed with ice cold PBS, then washed again with annexin V binding buffer (50 mM HEPES, 700 mM NaCl, 12.5 mM CaCl_2_, pH 7.4). Cells were resuspended in annexin V binding buffer plus annexin V-FITC (1∶20, Invitrogen) and incubated for 15 min. at room temperature. Later, cell suspensions were diluted with more annexin V binding buffer plus SYTOX Red (5 nM) and incubated for a further 15 min. Flow cytometry was then performed on a FACSCanto flow cytometer (BD Biosciences). A minimum of 10,000 cells were analyzed for every sample.

#### Assessment of caspase 3/7 activity, protease activity, and ATP cellular levels

HeLa cells were seeded at 7500 cells/well a day prior to experiments in white flat clear-bottom 96-well plates (Greiner Bio one). Cells were incubated with Cbl, Cbl conjugates, staurosporin or digitonin as appropriate at indicated concentrations. Cellular ATP levels and protease activity were measured after a 90 minute incubation using the Mitochondrial ToxGlo assay (Promega) as per manufacturer's instructions. To assess caspase 3/7 activity, cells were incubated with compound for 6 hours prior to measurement with the Caspase-Glo® 3/7 Assay (Promega) as per manufacturer's instructions.

#### Western blots

After treatment, cells were harvested and washed with ice cold PBS prior to lysis with RIPA buffer plus protease inhibitor cocktail (Santa Cruz Biotech) at 4°C for 20 min. Cells were then centrifuged at 16,000 g at 4°C for 10 minutes and the supernatant was collected. Total protein concentrations were quantified using the BCA assay and 40 μg of total protein in each sample was run on a 10% or 15% SDS-PAGE gels. Gels were transferred onto nitrocellulose or PVDF membranes and blocked with 5% BSA-TBST. Membranes were probed with primary antibody (1∶5000 β-actin antibody (Abcam), 1∶1000 PARP-1 antibody (Cell Signaling), 1∶1000 cleaved Caspase-3 antibody (Cell Signaling), 1∶5000 Biotin antibody (Jackson ImmunoResearch), 1∶1000 TBP antibody (Abcam) and 1∶1000 Histone H3 antibody (Abcam)). Membranes were then washed with TBST and incubated with 1∶5000 donkey anti-mouse (Santa Cruz Biotech) or goat anti-rabbit (Jackson ImmunoResearch) IgG-HRP secondary Ab for 1 hour at room temperature in 2% milk-TBST prior to chemiluminescence detection (GE Amersham).

#### Cbl-conjugate Labeling of mtDNA

HL-60 cells (25 million cells) were incubated with Cbl-TPP (10 uM) for 30 min, after which the cells were collected. The cells were then washed with ice-cold PBS, and their mitochondria were isolated using Mitochondrial Isolation Kit for Mammalian Cells (Thermo Scientific). The mtDNA was extracted from the mitochondrial pellets using AllPrep DNA/RNA Mini Kit (QIAGEN). DNA concentration was measured using a NanoDrop 1000 Spectrophotometer (Thermo Scientific) and then normalized (10 ng/μl). After this, a nitrocellulose membrane was spotted with untreated and treated DNA and UV crosslinked using UV Stratalinker 1800 (Stratagene) The membrane was blocked with 5% BSA-TBST for 1 hr at room temperature and then probed with TPP antibody (1∶5000) at 4°C overnight. Then the membrane was washed and visualized with chemiluminescence detection (GE Amersham).

#### Cbl-conjugate labeling of mitochondrial proteins

HL-60 cells (25 million cells) were incubated with biotin-labeled mt-Cbl peptide (Biotin-mt-Cbl) (0, 1.5 μM) or Cbl-TPP (10 uM) for 30 min, after which the cells were collected. The cells were then washed with ice-cold PBS, and their mitochondria were isolated using Mitochondrial Isolation Kit for Mammalian Cells (Thermo Scientific). The mitochondrial pellets were then lysed using RIPA buffer plus protease inhibitors for 30 min. at 4°C and the total protein concentration determined using BCA assay. Lysates (10 μg of total protein) were then loaded onto 7% gels and run at 30 V for 30 min. and 150 V for 1 hour using a tris-tricine buffer system (Cathode buffer: 0.1 M Tris, 0.1 M Tricine, and 0.1% SDS, Anode buffer: 0.2 M Tris-Cl, pH8.9). Immunoblotting was continued as described previously using biotin antibody to detect the biotin tag on mt-Cbl or TPP antibody to detect Cbl-TPP.

#### siRNA Knockdown of EXOG

HeLa cells were plated in a six well dish at a density of 150,000 cells/well one day prior to siRNA transfection. 10 nM Invitrogen Silencer Select siRNA against EXOG was transfected using RNAiMax Lipofectamine reagent (Invitrogen). 48 H after transfection cells were treated with a range of concentrations of mtCbl. Cell viability was determined 18 hours following treatment as described previously. Genomic DNA was extracted in parallel 48 H after transfection using a Genomic DNA Miniprep kit (Sigma) and quantified using Pico Green. Quantitative amplification of an 8.9 kb mitochondrial segment and a 17.7 kb b-globin target sequence was performed using the GeneAmp XL PCR kit (Perkin-Elmer) as described previously [17]. The presence of mitochondrial DNA lesions without significant nuclear DNA damage was used as a marker for successful knockdown of EXOG.

### *In vivo* Studies

#### Compound stability and hydrolysis in a biological environment

*Stability of mt-Cbl and MPP in mouse plasma:* Thiazole orange-labelled conjugates were incubated in mouse plasma for three weeks at room temperature. The stability of these compounds was then assessed via reverse-phase HPLC. A single peak with the identical retention time to a fresh conjugate control was considered to be a positive result for stability in mouse plasma. *Rate of mt-Cbl and Cbl hydrolysis in saline or plasma:* Cbl and mt-Cbl were incubated in saline or plasma for 0 h, 0.5 h, 1 h, 1.5 h, 2 h, 2.5 h and 3 h at 37°C. Following this, plasma proteins were precipitated in four volumes of ice-cold acetonitrile and centrifuged at 10,000×g for 10 min, 4°C. The resulting supernatant was analyzed via HPLC-MS/MS to determine active drug concentrations in each sample.

#### Maximal tolerated dose (MTD)

Non-obese diabetic/severe combined immunodeficiency (NOD/SCID) mice were treated intraperitoneally with increasing concentrations of Cbl (10–500 mg/kg) or mt-Cbl (10–50 mg/kg). Animals were monitored daily for weight loss, lethargy and other signs of physical discomfort. The MTD was determined to be the highest concentration of drug that did not result in moribundancy. All animal studies were carried out according to the regulations of the Canadian Council on Animal Care and with the approval of the Ontario Cancer Institute Animal Ethics Review board or the Faculty of Medicine Animal Care Committee at the University of Toronto.

#### Pharmacokinetic studies

Animal handling and dose administration was performed by Kard Scientific (Beverly, MA). Six CD1 mice were treated with a single dose of 10 mg/kg mt-Cbl or Cbl via intraperitoneal injection. Blood samples were collected at 15 min, 30 min, 1 h, 2 h, 4 h and 8 h in heparin-treated tubes. Samples were kept on ice until centrifugation at 1,500 rpm for 20 min, 4°C and the resulting plasma supernatants were transferred to fresh tubes. Plasma proteins were precipitated with four volumes of ice-cold acetonitrile and spun at 10,000×g for 10 min, 4°C. The resulting supernatant was analyzed via HPLC-MS/MS to determine plasma concentrations of Cbl and mt-Cbl. Drug elimination profiles, C_max_ and T_1/2_ were also obtained from these data.

#### Biodistribution studies

K562 cells (3×10^6^) were injected subcutaneously into the flanks of ten NOD/SCID mice (Ontario Cancer Institute, Toronto, ON). Palpable tumours were formed 7 days after injection and mice were then treated daily for 10 days with Cbl (100 mg/kg) or mt-Cbl (15 mg/kg) by intraperitoneal injection. Mice were sacrificed 17 days after injection of cells and organs were excised for analysis of Cbl and mt-Cbl biodistribution profiles. The liver, kidneys, spleen, heart, lungs and brain were removed from mice following sacrifice and immediately frozen in dry ice until analysis. Tissues were homogenized in four volumes of ice-cold acetonitrile and centrifuged at 10,000×g for 10 min, 4°C. The resulting supernatant was analyzed via HPLC-MS/MS to determine drug concentrations in each tissue.

#### Infusion pump administration of mt-Cbl

OCI-AML2 cells (1×10^6^) were injected subcutaneously into the flanks of ten NOD/SCID mice (Ontario Cancer Institute, Toronto, ON). The following day, animals were randomly assigned to two groups and implanted with an infusion pump containing either mt-Cbl (2.5 mg/kg) or vehicle control. The pump delivered its contents in a 24 h continuous infusion manner at a rate of 0.5 μL/h. Tumor volumes were measured three times a week using calipers. Mice were sacrificed 14 days after injection of cells, tumors excised and their volume and mass measured.

#### Assessment of cellular toxicity in xenograft tumors and mouse livers

Mice were treated every other day with equimolar doses of either mt-Cbl (15 mg/kg), Cbl (3.7 mg/kg) or vehicle control for 21 days by Kard Scientific (Beverly, MA). Following sacrifice, mouse livers and K562 xenograft tumors were fixed in 10% formalin overnight and then transferred to 70% ethanol. Samples were then paraffin-embedded, sectioned and stained in a standard manner by the Pathology Research Program Laboratory at Toronto General Hospital. Biotin-conjugated secondary antibodies, avidin horseradish peroxidase and the chromogenic diaminobenzidine tetrahydrochloride (DAB) were used for detection. Slides were imaged at 20× and then analyzed using Aperio ImageScope Software (Aperio Technologies, Vista, California). Positive pixels were identified and counted using the Positive Pixel Count V9 algorithm in this software. Quantification was performed on ten representative squares of 2500×2500 μm each and an average was calculated. % Positive Pixels was determined by dividing the positive pixel count by the total number of pixels analyzed [18]. A standard hematoxylin and eosin (H&E) staining was performed on liver sections and these were reviewed for hepatotoxicity and injury by a pathologist with liver expertise. In addition, liver toxicity was evaluated though determination of levels of specific liver enzymes. Mice were treated every other day with equimolar doses of either mt-Cbl (15 mg/kg), Cbl (3.7 mg/kg) or vehicle control for 21 days. Three days after the final dose, mouse plasma samples were tested for levels of bilirubin, alkaline phosphatase and aspartate transaminase in a standard manner.

## Results and Discussion

### Establishing the intramitochondrial target of mt-Cbl

In a previous study [11], we demonstrated that mt-Cbl was able to crosslink mtDNA and induce DNA lesions *in vitro* and we hypothesized that this form of damage resulted in the activation of cell death. However, Cbl is a promiscuous alkylating agent and could potentially bind to various nucleophilic targets other than DNA such as proteins or high abundance metabolites. In this study, we sought to understand the importance of DNA damage relative to other biochemical events.

**Figure 2:**
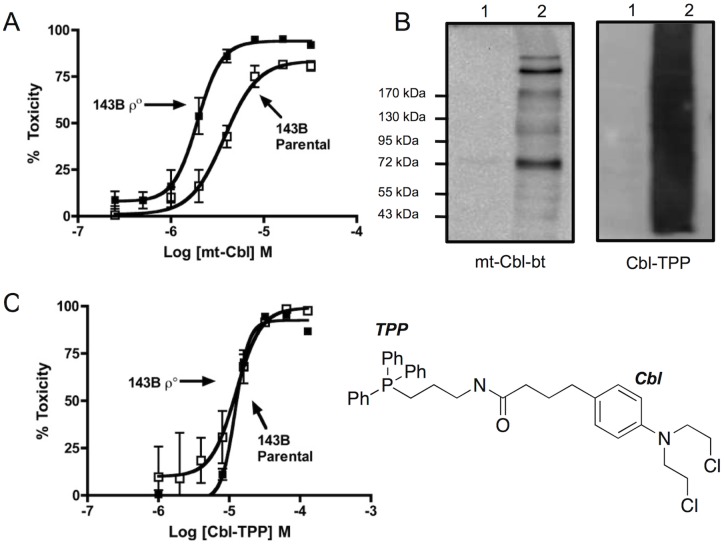
Assessment of the role of protein damage in mt-Cbl cytotoxicity. (a) ***Toxicity of mt-Cbl in a*** ρ° ***cell model.*** After overnight incubation, mt-Cbl was more potent in the ρ° cell line indicating the importance of mt-Cbl's protein targets. (b) ***Mt-Cbl and Cbl-TPP mitochondrial protein targets in vitro.*** HL60 cells were treated with mt-Cbl-bt or Cbl-TPP and then mitochondrial lysates were immunoblotted. Lane 1: Control HL60 cells, Lane 2: Treated HL60 cells. (c) ***Toxicity of Cbl-TPP in a*** ρ ° ***cell model.*** Cbl-TPP toxicity profiles in 143B parental and its ρ° derivative overlap.

We tested whether mt-Cbl toxicity was mainly due to mtDNA lesions by testing a cell line that lacks this genome. Toxicity of mt-Cbl in the osteosarcoma 143B cell line and its mtDNA-free ρ° derivative that was confirmed to be depleted of DNA (see supplementary information for Figure S1) was assessed. Incubation of both cell lines with various doses of mt-Cbl overnight resulted in surprisingly similar toxicity profiles (Figure 2A). These results indicate that it is not only alkylation of mtDNA by mt-Cbl that plays a crucial role in inducing cell death.

To ensure that the non-DNA based origin of toxicity did not stem from the presence of the peptide delivery vector, we tested the requirement for the alkylating agent and for the specific delivery peptide used. Testing of mt-Cbl after overnight deactivation in phosphate buffered saline indicated that the alkylation activity of the compound is necessary for its biological activity (see supplementary information for Figure S2). The deactivated mt-Cbl did not show any appreciable toxicity up to the highest concentration tested (32 µM). In addition, we tested the impact of mitochondrial localiztion on the activity of Cbl. Using a delivery vector replacing the hydrophobic cyclohexylalanies with tyrosines, a Cbl-peptide conjugate can be directed to accumulate within the nucleus and cytoplasm of cells rather than the mitochondrion (see supplementary information for Figure S3). This compound was found to have a greatly reduced activity compared to the mitochondrial targeted version, mt-Cbl. Thus the activity of mt-Cbl is directly dependant on alkyation of a target within the mitochondrion.

Having confirmed that mitochondria-specific alkylation damage was implicated in the toxicity observed, we therefore sought to test whether mtDNA damage was being repaired within the mitochondrion and leading to similar toxicity between the ρ° derivative and WT cell line. We thus hypothesized that reducing mitochondrial base excision repair (BER) activity might potentiate the toxicity of mt-Cbl. Unlike nuclei which possess multiple and varied pathways for repairing DNA damage, mitochondria repair their DNA mostly through base excision repair [19]. This pathway is known to be critical for the maintenance of mtDNA integrity in the presence of endogenous ROS. HeLa cells were transfected with siRNA against EXOG, a mitochondrial exonuclease that has been shown to be essential for mitochondrial base excision repair [20]. Knockdown of EXOG has been shown in other studies to cause accumulation of oxidative lesions in mtDNA. To confirm successful knockdown of EXOG expression a PCR amplification assay was used to detect mtDNA damage. Indeed significant and specific mtDNA damage was observed in the EXOG siRNA transfected control 48 hours after transfection (see supplementary information for Figure S4). Inactivation of mitochondrial BER, however, failed to affect the toxicity of mt-Cbl. These results indicate that mt-Cbl induced DNA lesions are not repaired by BER and provide further evidence that mt-Cbl toxicity is not mediated by DNA damage.

The mitochondrion is densely packed with proteins that play crucial functions in regulating overall cellular homeostasis. Some of these proteins are involved in the production of ATP, such as the ATP synthase, while others are involved in activating the intrinsic cell death pathway, such as the permeability transition pore complex (PTPC) [21]. If mt-Cbl bound to such proteins, the damage induced could lead to enhanced cellular toxicity due to the vital role these proteins play within a cell [22]. To visualize possible protein targets of mt-Cbl, we performed western blots on the mitochondrial lysates of HL60 cells treated with a biotin-labeled version of mt-Cbl (bt-mt-Cbl). The results obtained revealed that mt-Cbl did alkylate mitochondrial proteins at significant levels (Figure 2B). Interestingly, specific bands were repeatedly isolated, indicating there there may be some specifcity in the alkylation, but repeated attempts to identify individual proteins were unsuccessful, indicating that a very heterogeneous mix of proteins are likely reacting.

### Exploring the generality of mitochondrial protein alkylation

To investigate whether a DNA-independent mechanism of cell death would be observed for a different form of mitochondrially-targeted Cbl delivered using a different vector, we designed and synthesized a drug that uses the triphenylphosphonium (TPP) ion for delivery. TPPs have been extensively studied and used to successfully deliver therapeutic cargo to mitochondria [23]. Characterization of Cbl-TPP revealed that TPP conjugation produced a compound that retained alkylation activity very similar to that observed with the MPP conjugated form of mt-Cbl (see supplementary information for Figure S5).

The reactivity of Cbl-TPP with protein targets was assessed in HL-60 cells. After incubation of the cells with drug, the mitochondria were isolated and lysed. Protein and DNA adducts were then visualized using immunoblotting with an anti-TPP antibody. Alkylation of both mtDNA and proteins (Figure S5; Figure 2B) was observed. Interestingly, Cbl-TPP bound to mitochondrial proteins with less specificity than mt-Cbl, indicating that the peptide portion of mt-Cbl may in fact impart some degree of specificity.

To assess whether the DNA damage caused by Cbl-TPP was a cause of cell death, we again used the DNA-free ρ° model. Treatment of 143B cells and a ρ° derivative with Cbl-TPP revealed overlapping toxicity profiles (Figure 2C) very similar to those observed with mt-Cbl. Therefore, the activity of Cbl-TPP is also not linked to DNA alkylation, and the shift in lethal target appears to be a general effect for mitochondria-targeted chlorambucil.

### Cell death pathway of mitochondria-targeted chlorambucil

Since redirecting Cbl to the mitochondrion alters the drug's main target, we sought to investigate if this results in a different cell death pathway being activated. DNA damaging agents like Cbl are known to activate the intinsic or mitochondrial cell death pathway [24]. This results in the release of cytochrome c from the inner mitochondrial membrane into the cytoplasm and the assembly of the apoptosome, which activates caspase-3 and caspase-7 [25]. These two caspases are then responsible in executing apoptosis and cleaving various critical cellular components such as poly (ADP-ribose) polymerase-1 (PARP-1) [25].

**Figure 3:**
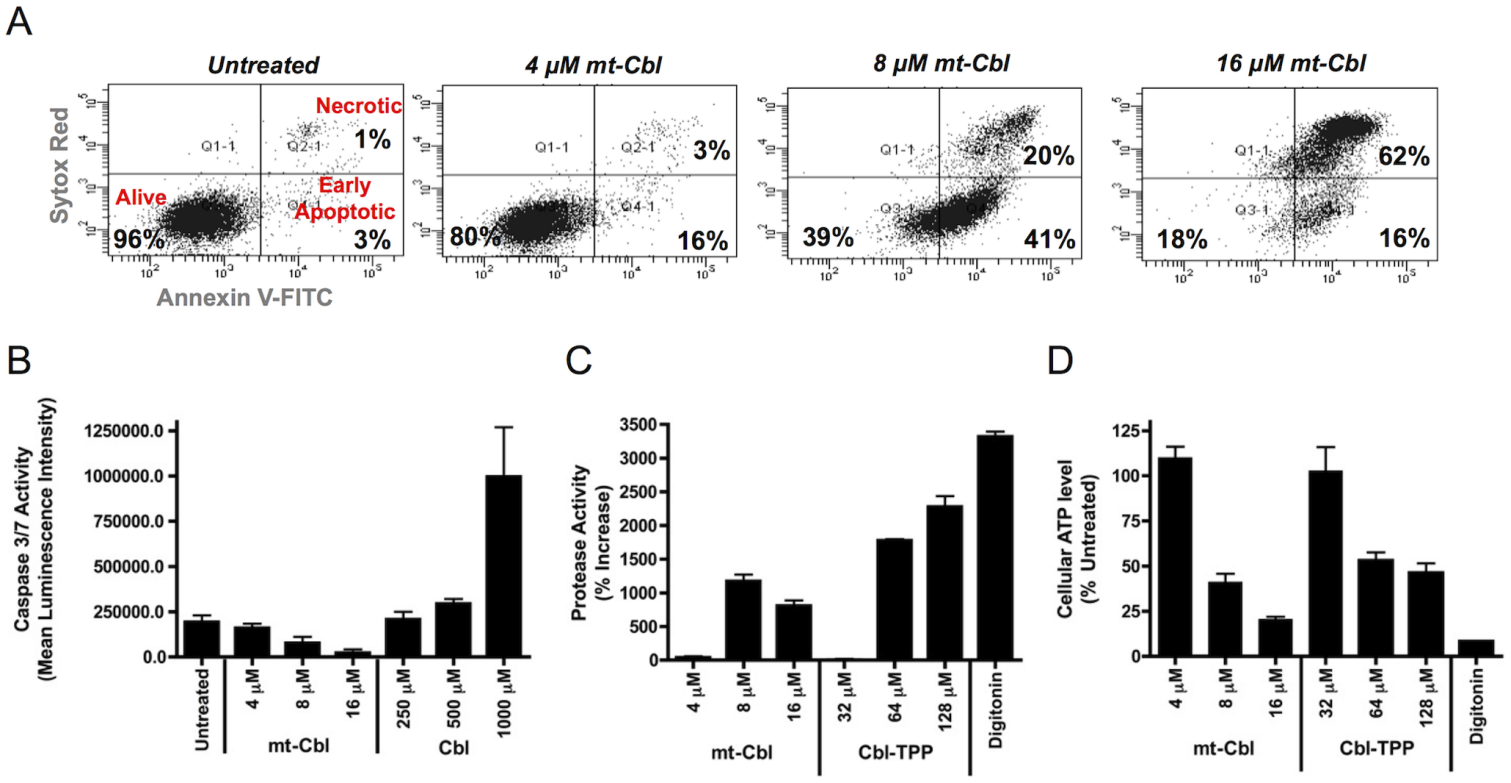
Determination of the cell death mechanism of mt-Cbl. (a) ***FACS analysis of HeLa annexin V-FITC*** (***A-FITC***) ***and SYTOX red*** (***SR***) ***cell staining after mt-Cbl treatment for 90*** ***min.*** (A-FITC-/SR-, alive; FITC+/SR-, early apoptotic; A-FITC+/SR+, necrotic). Percentage of cells in each quadrant is indicated. Mt-Cbl treatment causes necrotic cell death in a dose-dependent manner. (b) ***Caspase-3/7 activity in HeLa cells.*** Treatment of HeLa cells with Cbl for 6 hours results in an increase in caspase-3/7 activity in a dose-dependent manner, while mt-Cbl has no effect. Mean values plotted, n = 4, error bars are standard deviation. (c) ***Protease activity in treated HeLa cells.*** Treatment of HeLa cells with mt-Cbl or Cbl-TPP at concentrations over EC50 results in an increase in protease activity. Mean values plotted, n = 3, error bars are s.e.m. (d) ***Cellular ATP level in treated HeLa cells.*** Treatment of HeLa cells with mt-Cbl or Cbl-TPP at concentrations over EC50 results in a dramatic drop in cellular ATP levels. Mean values plotted, n = 3, error bars are s.e.m.

We tested the caspase-dependency of cell death induced by Cbl and mt-Cbl using a caspase-3/7 activity assay. Cbl treatment of HeLa cells resulted in an increase in caspase-3/7 activity in a dose-dependent manner, while mt-Cbl treatment had no effect (Figure 3B). These results were further validated through immunoblotting where Cbl induced the cleavage and activation of caspase-3 (13 and 19 kDa, active forms of caspase-3) and the cleavage of PARP-1 (89 kDa) (see supplementary information for Figure S6). Pretreatment with a pan-caspase inhibitor, Q-VD-Oph, suppressed the effects of Cbl treatment. No activated caspase-3 fragments or cleaved PARP-1 fragments were noted upon mt-Cbl treatment.

In our previous study, flow cytometry analysis of Annexin V-FITC (A-FITC)/SYTOX Red (SR) cell staining after mt-Cbl overnight treatment showed a significant increase in the percentage of A-FITC+/SR+ cells [11]. Due to the long drug incubation time, these cells could be labeled as either late apoptotic or necrotic. To elucidate the difference, mt-Cbl toxicity was measured over a short time interval (90 min.) in HeLa cells (Figure 3A). The percentage of A-FITC+/SR+ cells was found to increase in a dose-dependent manner. This indicated that mt-Cbl treatment resulted in the loss of plasma membrane integrity and the activation of necrosis. To further validate this finding, protease activity and ATP cellular level assays were employed. Treatment of HeLa cells with mt-Cbl and Cbl-TPP at various doses for 90 minutes resulted in a significant increase in protease activity and a drastic drop in ATP cellular levels (Figure 3C and D). Taken together, these findings reveal that redirecting Cbl to the mitochondria does not only alter the drug's main target but also results in a shift in the cell death mechanism from apoptosis to necrosis. This change in the cell death mechanism explains why mt-Cbl was able to bypass drug resistance mechanisms that suppress the toxicity of the parental drug [11]. The majority of cancer drug resistance pathways block apoptotic cell death induced by current chemotherapeutics. However, no resistance pathway has been discovered, as of yet, that can block a necrotic signal. Recent studies have shown that by taking advantage of necrosis, apoptosis-resistant tumor cells could be eliminated [26]–[28].

### *In vivo* pharmacokinetic and biodistribution profile of mt-Cbl

Having investigated the effect of mitochondrial rerouting on Cbl's efficacy and mechanism of action *in vitro,* we wanted to determine whether mt-Cbl would exhibit efficacy within an *in vivo* model, and pursued the pharmacokinetic characterization of the drug to assess its stability *in vivo*. Initially, we assessed the stability of the peptide vector in mouse plasma after a three-week incubation period using high performance liquid chromatography (HPLC) and found the peptide to be stable (see supplementary information for Figure S7). This is likely because the MPP vector consists of synthetic cyclohexylalanine and *D*-arginine monomers, which cannot be degraded by proteases. Because Cbl is highly susceptible to hydrolysis in aqueous conditions, such as water and blood, we also assessed the half-life of Cbl and mt-Cbl in saline and plasma and found comparable half-lives of 1 hour for both of these compounds (see supplementary information for Figure S8). These values were similar to the Cbl half-life reported in the literature, which is 30 minutes in water and 45 minutes in blood [9], [29].

**Figure 4:**
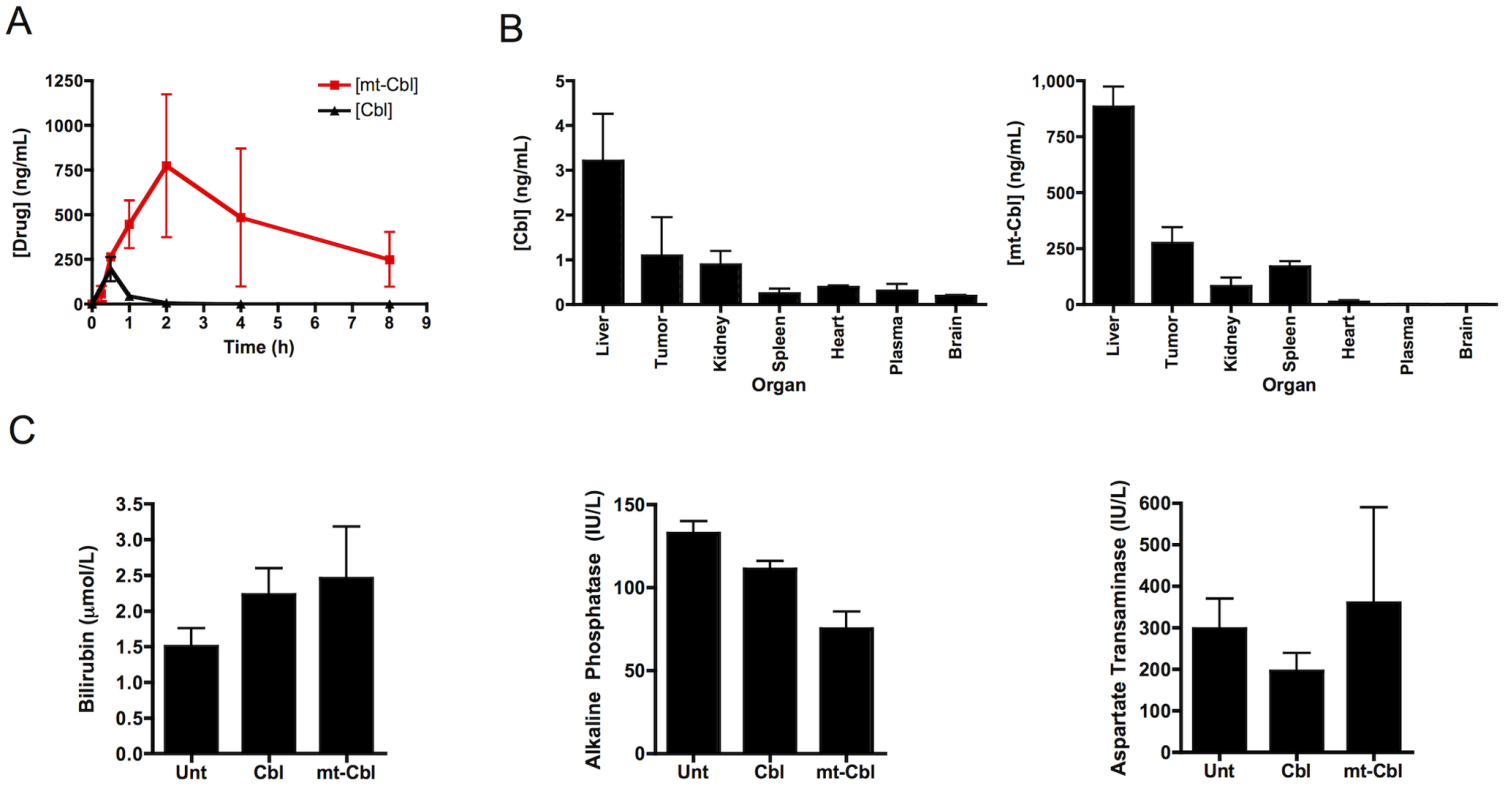
*In vivo* profiles of mt-Cbl and Cbl. (a*)* ***Pharmacokinetic profile of mt-Cbl and Cbl.*** 10 mg/kg of Cbl or mt-Cbl was administered to mice and plasma levels of both compounds were measured via HPLC-MS/MS. Mt-Cbl is eliminated more gradually than Cbl. Mean values plotted, n = 3, error bars are standard deviation. (b*)* ***Biodistribution of mt-Cbl and Cbl.*** Both compounds show similar profiles suggesting that the MPP peptide does not alter distribution of Cbl. Mean values plotted, n = 3, error bars are standard deviation. (c) ***Liver enzyme levels following treatment.*** Mice were treated as above and their plasma was assessed for levels of bilirubin, alkaline phosphatase and aspartate transaminase. No significant increase was noted in any of the enzyme levels following treatment with Cbl or mt-Cbl. Mean values plotted, n = 4, error bars are s.e.m.

Since biological activity is dependent on the drug being efficiently absorbed into the blood, we then evaluated the pharmacokinetic profile of mt-Cbl compared to Cbl in mice using high performance liquid chromatography – mass spectrometry (HPLC-MS/MS). We observed that mt-Cbl had a higher C_max_ and a larger AUC compared to Cbl (Figure 4A and Table 1). This enhanced absorption and prolonged retention has also been noted for cargo delivered by cell penetrating peptides (CPPs) [30], [31].

**Table 1:** Pharmacokinetic parameters for mt-Cbl and Cbl.

	Cbl	Mt-Cbl
T_1/2_	45 min	6 h
C_max_	200 ng/mL	800 ng/mL
AUC	147	3562

An improved pharmacokinetic profile would likely result in increased uptake of the drug into tissues. Indeed, biodistribution studies showed enhanced mt-Cbl permeation with an ∼250-fold greater accumulation of mt-Cbl in tumors, compared to Cbl (Figure 4B). Despite this enhanced accumulation, both drugs displayed a similar tissue distribution profile. This consistency in tissue distribution has also previously been noted in CPPs delivery studies [29], [30]. The liver was the site of greatest accumulation for both mt-Cbl and Cbl and very low levels of both drugs were detected in the kidneys. This is also supported by previous reports that Cbl is primarily metabolized by the liver and has low elimination through the kidneys [32].

In order to ensure that these elevated levels of mt-Cbl were not hepatotoxic, due to mt-Cbl-mediated necrosis, a standard H&E stain was performed and assessed by a pathologist. No hepatoxicity or liver injury was observed and the sections were found to be histologically normal (see supplementary information for Figure S9). Hepatotoxicity was also assessed in terms of alterations in plasma levels of bilirubin and liver enzymes alkaline phosphatase (ALP) and aspartate aminotransferase (AST). No significant change was noted in the levels of all three indicators of hepatic damage upon Cbl or mt-Cbl treatment (Figure 4C). Increased drug accumulation in the liver without increased toxicity has been observed in other drug-targeting studies [33].

### *In vivo* toxicity and efficacy of mt-Cbl

**Figure 5:**
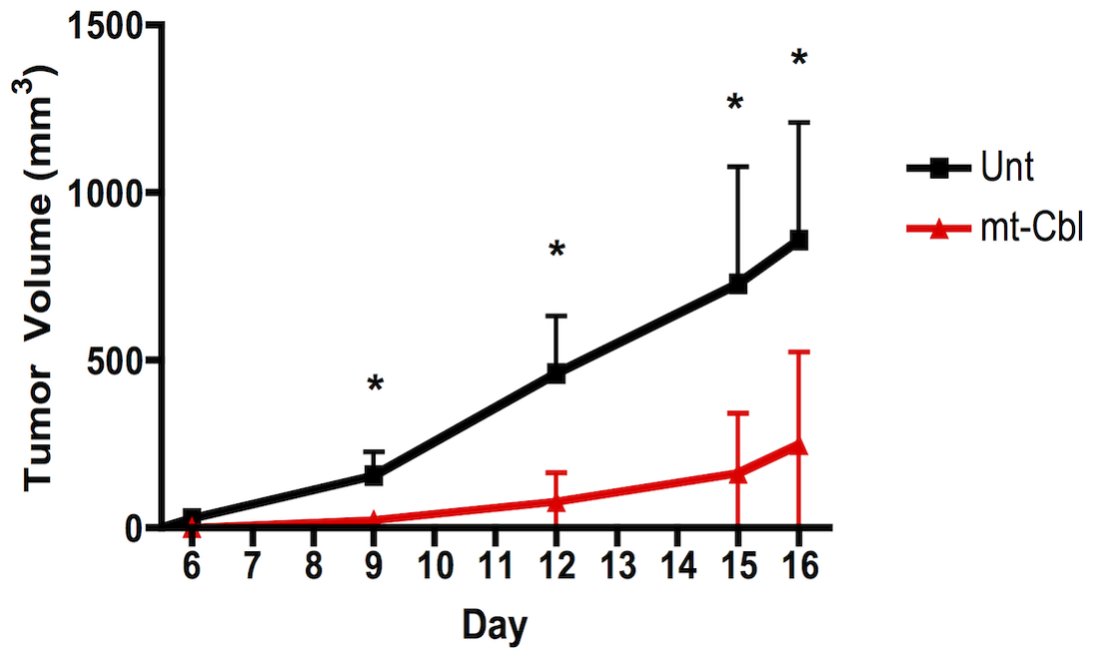
*In vivo* efficacy of mt-Cbl. (a) ***Tumor growth following infusion pump administration of mt-Cbl.*** Mice were treated with a continuous infusion of 2.5 mg/kg of mt-Cbl and tumor volumes were compared to untreated mice. Mean values plotted, n = 8, error bars are standard deviation, *P<0.05.

Next, we sought to investigate whether mt-Cbl could inhibit tumor growth *in vivo*, in a leukemia xenograft mouse model. A pilot study performed to determine the maximal tolerated dose (MTD) of Cbl and mt-Cbl found these values to be 400 mg/kg and 50 mg/kg, respectively. We then assessed the anti-cancer activity of mt-Cbl in an AML xenograft model. Animals were injected with OCI-AML2 cells and the following day, an infusion pump loaded with mt-Cbl or vehicle control was implanted. This allowed the drug to be delivered in a continuous manner, rather than in bolus doses, and its ability to delay tumor growth was assessed. Animals treated with mt-Cbl exhibited a statistically significant delay in tumor formation compared to untreated animals (Figure 5). In addition, the relative tumor volume of treated mice divided by that of the control mice (T/C value [34]) was 29% with infusion compared to 61% with bolus injection. This enhanced anti-tumor activity with prolonged infusion has also been noted for other chemotherapeutic agents [34].

## Conclusions

This work has allowed further characterization of the first DNA alkylating agent to be retargeted to the mitochondria and the demonstration of its *in vivo* efficacy. We show that the mitochondrial targeting of Cbl not only changes its intracellular site of action, but also shifts its primary target under the conditions studied from DNA damage to protein damage. This change in the main drug target translated into a different, necrotic cell death pathway being activated. Our *in vivo* studies revealed that drug targeting with a MPP results in improved absorption and retention of the compound and a similar biodistribution profile compared to the parent drug. In addition, the mitochondria targeted alkylating agent showed encouraging anti-cancer activity *in vivo* with no drug-induced toxicity within its therapeutic window. Further work needs to be done to assess whether there are structural changes to the drug or peptide that could allow this molecule to more selectively target mitochondrial DNA.

## Supporting Information

Figure S1 **Respiration rate of 143B parental** (**ρ^+^**) **and ρ**° **cell lines.** The O_2_ consumption of 2,000,000 cells (ρ^+^ or ρ°) was measured over several time intervals and the rate of O_2_ consumption was calculated. Mean values plotted, n = 2, error bars are standard deviation.
(PDF)

Figure S2 **Effect of Cbl deactivation on mt-Cbl toxicity in HeLa cells.** mt-Cbl was incubated overnight in phosphate-based buffer (PBS) to deactivate Cbl. HeLa cells were treated with various doses of deactivated mt-Cbl overnight. Toxicity of mt-Cbl was drastically attenuated indicating that mt-Cbl's toxicity is due to Cbl's alkylation activity and not the peptide vector.
(PDF)

Figure S3 **Cytoplasmic and Nuclear targeted Cbl is less potent than mt-Cbl.** (a) Chemical structure of nuclear-targeted Cbl (n-Cbl). Replacement of cyclohexylalanines in the MPP with tyrosines results in nuclear targeting of the Cbl-peptide conjugate. (b) Cellular Localization of thiazole orange labeled nuclear-targeted peptide (n-pep) in live HeLa cells compared with DAPI. The fluorescence signal shows predominantly nuclear and cytoplasmic staining. (c) Cellular uptake of to-labeled n-pep and mt-pep in HeLa cells using flow cytometry. The n-pep exhibits equal levels of uptake relative to the mt-pep at double the concentration. (d) Toxicity profiles of n-Cbl and mt-Cbl in live HeLa cells following overnight incubation. mt-Cbl is more potent that n-Cbl at concentrations with equal cellular uptake.
(PDF)

Figure S4 **Determination of mitochondrial capacity for repair of mt-Cbl induced DNA lesions.** (a) **Toxicity of mtCbl in cells treated with siRNA against EXOG** (**siEXOG**)**.** Knockdown of mitochondrial BER has no effect on mt-Cbl toxicity, indicating that DNA damage is not being repaired by this pathway. siNT is the untargeted siRNA control. LD_50_ for mt-Cbl after siNT or siEXOG transfection was 3.5 μM and 4.7 μM, respectively. (b) **Mitochondrial and nuclear DNA lesions induced by EXOG knockdown.** A significant reduction in PCR amplification of the mitochondrial genome is observed 48 h after transfection of siEXOG compared to the non-targeted siRNA control indicating successful knockdown of EXOG. No reduction in PCR amplification of the nuclear genome is seen 48 h after transfection, demonstrating the specificity of EXOG knockdown.
(PDF)

Figure S5 **Characterization of Cbl-TPP.** (a) ***Chemical structure of Cbl-TPP*** **.** Cbl was conjugated to a triphenylphosphonium ion to generate Cbl-TPP. (b) ***Alkylation activity of Cbl, mt-Cbl and Cbl-TPP.*** Rate of alkylation of Cbl and its conjugates was calculated after measuring the absorbance of 4-(4-(Nitrobenzyl)pyridine upon Cbl alkylation. Conjugation of Cbl to mitochondria-targeting vectors reduces its alkylation activity. Mean values plotted, n = 3, error bars are s.e.m. (c) **Alkylation of mtDNA in vitro.** HL60 cells were incubated with Cbl-TPP and then their mtDNA was isolated. Untreated (left) and treated mtDNA (right) was probed with TPP antibody to assay for Cbl-TPP-DNA adducts. A positive signal indicates the presence of Cbl-TPP DNA adducts.
(PDF)

Figure S6 **Activation of caspase-3 and PARP-1 cleavage in treated HeLa cells.** Cbl treatment (750 μM) for 8 hours resulted in the cleavage and activation of caspase-3 (fragments 13 and 19 kDa), and the cleavage of PARP-1 (116 kDa to 85 kDa) by active caspases. Pretreatment of a pan-caspase inhibitor, Q-VD-Oph (20 μM), suppresses the effects of Cbl. Treatment with mt-Cbl has no effect.
(PDF)

Figure S7 **Stability of mt-Cbl conjugate.** The conjugate was incubated for three weeks in mouse plasma at 37°C. Its purity was then assessed using HPLC and one peak was noted, suggesting that the conjugate was stable in mouse plasma.
(PDF)

Figure S8 **Half-life of mt-Cbl and Cbl.** The mt-Cbl and Cbl compounds were incubated for varying times in saline or mouse plasma at 37°C. The percentage of active drug was monitored via HPLC-MS/MS. Both compounds showed comparable T1/2 of 1 h in saline and plasma.
(PDF)

Figure S9 ***Immunohistochemistry of mouse livers following treatment.*** H&E staining was performed in a standard manner on livers from untreated mice or those treated with equimolar doses of Cbl (3.7 mg/kg) and mt-Cbl (15 mg/kg). All livers were histologically normal with no features of injury or toxicity. Images were reviewed by a pathologist with liver expertise. Representative images are shown above. Scale bar, 100 μm.
(PDF)
