# Supplementary figures and images for: Re-Directing an Alkylating Agent to Mitochondria Alters Drug Target and Cell Death Mechanism
Source: PLoS One. 2013 Apr 9;8(4):e60253. doi: 10.1371/journal.pone.0060253 (PMC3621862; doi:10.1371/journal.pone.0060253)

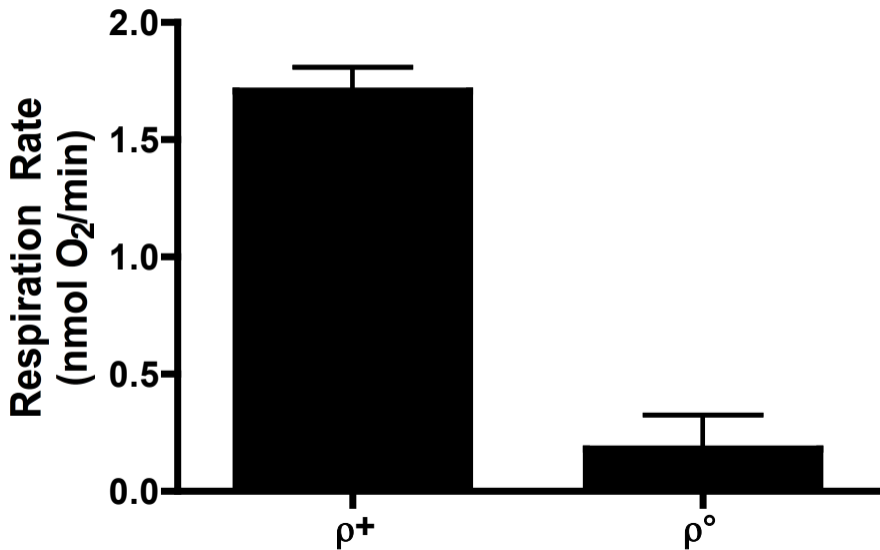

Supplement: Figure S1 — Respiration rate of 143B parental (ρ+) and ρ° cell lines. The O2 consumption of 2,000,000 cells (ρ+ or ρ°) was measured over several time intervals and the rate of O2 consumption was calculated. Mean values plotted, n = 2, error bars are standard deviation. (PDF) [file pone.0060253.s001.pdf]

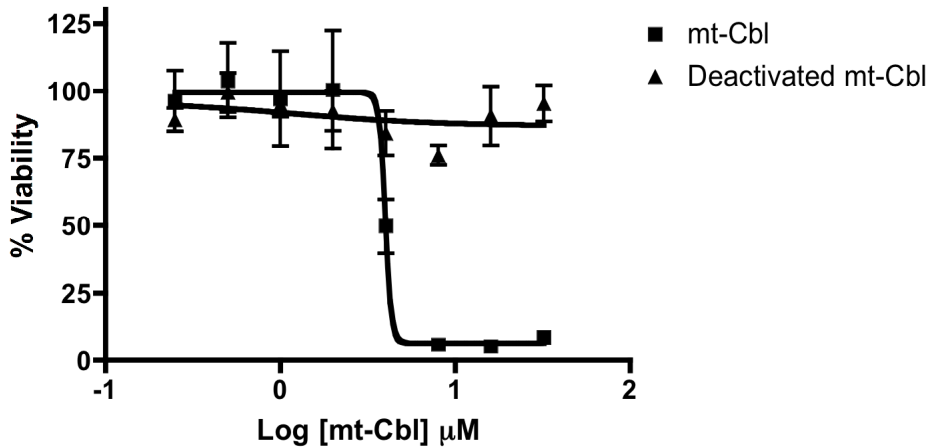

Supplement: Figure S2 — Effect of Cbl deactivation on mt-Cbl toxicity in HeLa cells. mt-Cbl was incubated overnight in phosphate-based buffer (PBS) to deactivate Cbl. HeLa cells were treated with various doses of deactivated mt-Cbl overnight. Toxicity of mt-Cbl was drastically attenuated indicating that mt-Cbl's toxicity is due to Cbl's alkylation activity and not the peptide vector. (PDF) [file pone.0060253.s002.pdf]

a

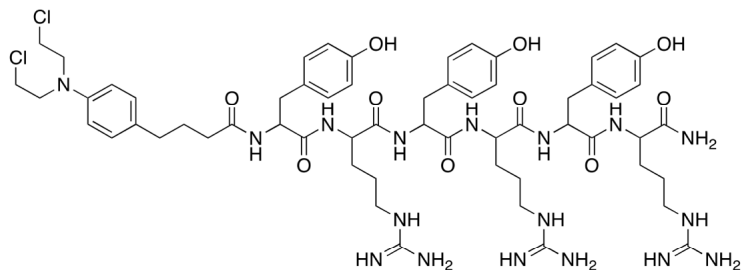

b

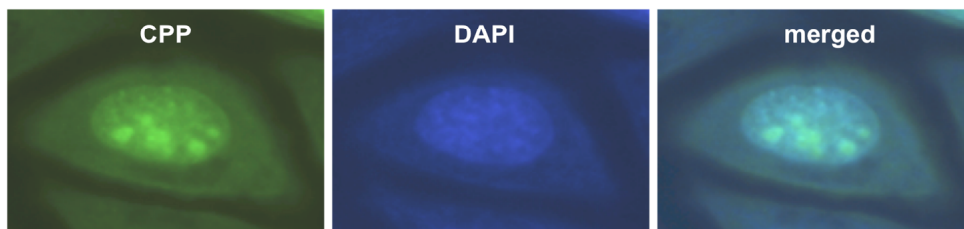

c

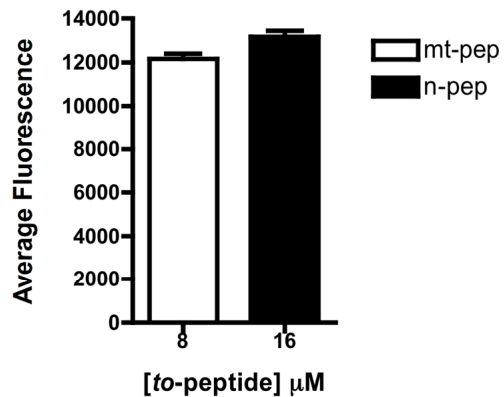

d

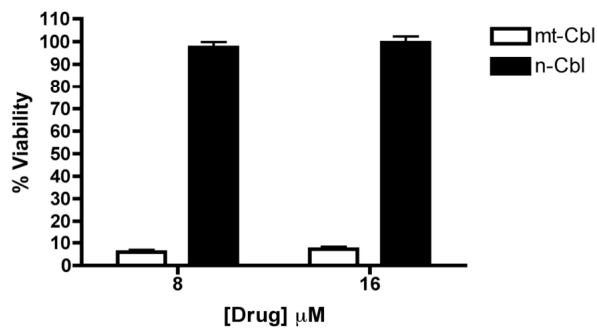

Supplement: Figure S3 — Cytoplasmic and Nuclear targeted Cbl is less potent than mt-Cbl. (a) Chemical structure of nuclear-targeted Cbl (n-Cbl). Replacement of cyclohexylalanines in the MPP with tyrosines results in nuclear targeting of the Cbl-peptide conjugate. (b) Cellular Localization of thiazole orange labeled nuclear-targeted peptide (n-pep) in live HeLa cells compared with DAPI. The fluorescence signal shows predominantly nuclear and cytoplasmic staining. (c) Cellular uptake of to-labeled n-pep and mt-pep in HeLa cells using flow cytometry. The n-pep exhibits equal levels of uptake relative to the mt-pep at double the concentration. (d) Toxicity profiles of n-Cbl and mt-Cbl in live HeLa cells following overnight incubation. mt-Cbl is more potent that n-Cbl at concentrations with equal cellular uptake. (PDF) [file pone.0060253.s003.pdf]

A

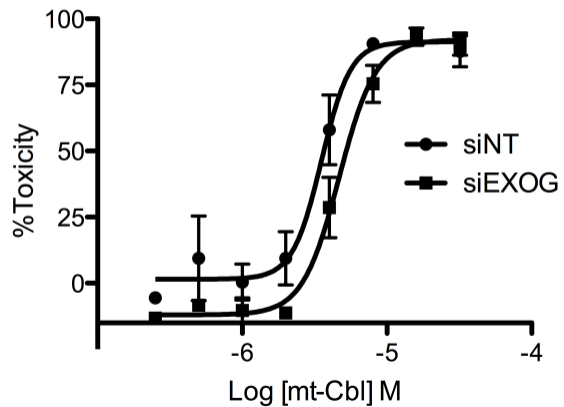

B

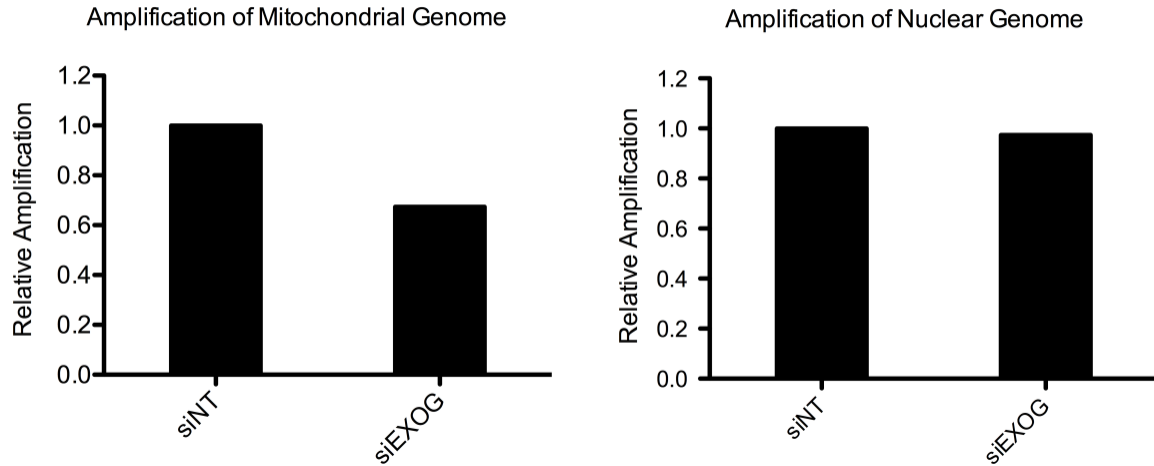

Supplement: Figure S4 — Determination of mitochondrial capacity for repair of mt-Cbl induced DNA lesions. (a) Toxicity of mtCbl in cells treated with siRNA against EXOG (siEXOG). Knockdown of mitochondrial BER has no effect on mt-Cbl toxicity, indicating that DNA damage is not being repaired by this pathway. siNT is the untargeted siRNA control. LD50 for mt-Cbl after siNT or siEXOG transfection was 3.5 μM and 4.7 μM, respectively. (b) Mitochondrial and nuclear DNA lesions induced by EXOG knockdown. A significant reduction in PCR amplification of the mitochondrial genome is observed 48 h after transfection of siEXOG compared to the non-targeted siRNA control indicating successful knockdown of EXOG. No reduction in PCR amplification of the nuclear genome is seen 48 h after transfection, demonstrating the specificity of EXOG knockdown. (PDF) [file pone.0060253.s004.pdf]

A

TPP

Cbl

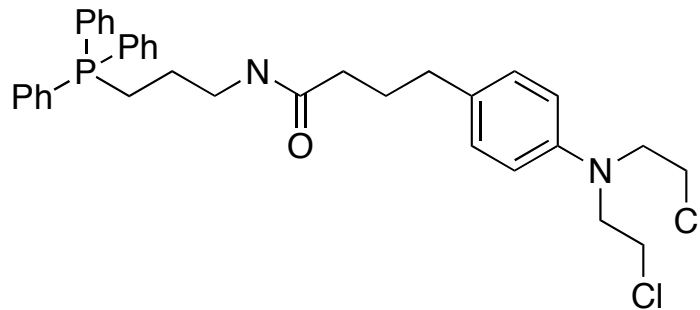

B

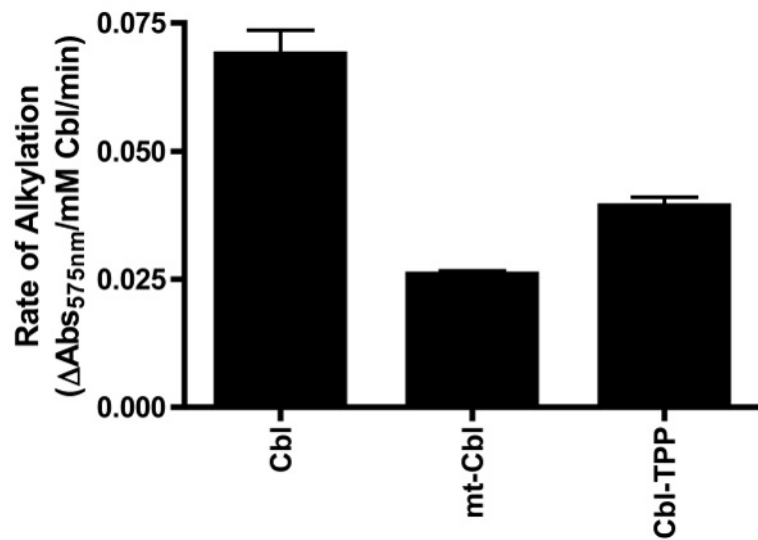

C

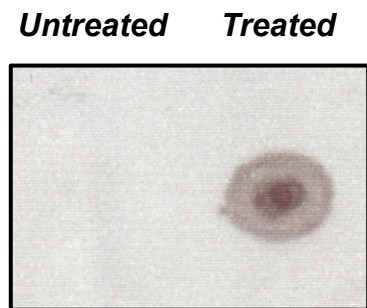

Supplement: Figure S5 — Characterization of Cbl-TPP. (a) Chemical structure of Cbl-TPP . Cbl was conjugated to a triphenylphosphonium ion to generate Cbl-TPP. (b) Alkylation activity of Cbl, mt-Cbl and Cbl-TPP. Rate of alkylation of Cbl and its conjugates was calculated after measuring the absorbance of 4-(4-(Nitrobenzyl)pyridine upon Cbl alkylation. Conjugation of Cbl to mitochondria-targeting vectors reduces its alkylation activity. Mean values plotted, n = 3, error bars are s.e.m. (c) Alkylation of mtDNA in vitro. HL60 cells were incubated with Cbl-TPP and then their mtDNA was isolated. Untreated (left) and treated mtDNA (right) was probed with TPP antibody to assay for Cbl-TPP-DNA adducts. A positive signal indicates the presence of Cbl-TPP DNA adducts. (PDF) [file pone.0060253.s005.pdf]

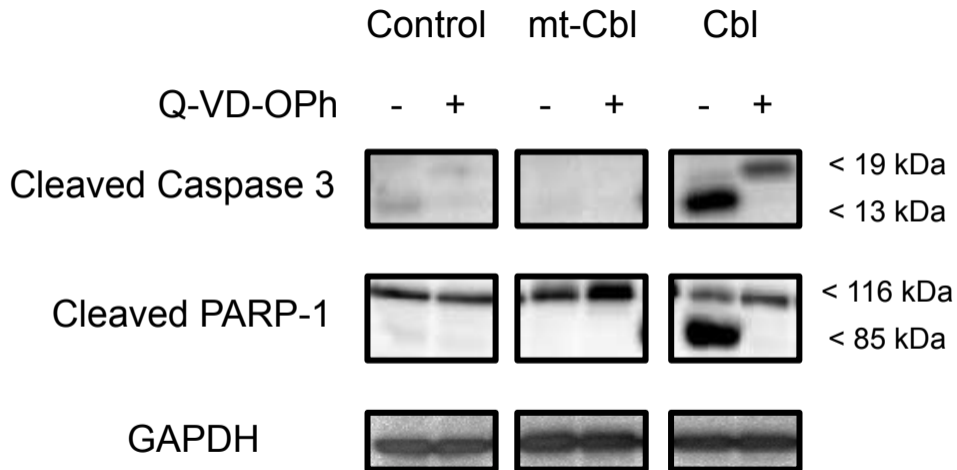

Supplement: Figure S6 — Activation of caspase-3 and PARP-1 cleavage in treated HeLa cells. Cbl treatment (750 μM) for 8 hours resulted in the cleavage and activation of caspase-3 (fragments 13 and 19 kDa), and the cleavage of PARP-1 (116 kDa to 85 kDa) by active caspases. Pretreatment of a pan-caspase inhibitor, Q-VD-Oph (20 μM), suppresses the effects of Cbl. Treatment with mt-Cbl has no effect. (PDF) [file pone.0060253.s006.pdf]

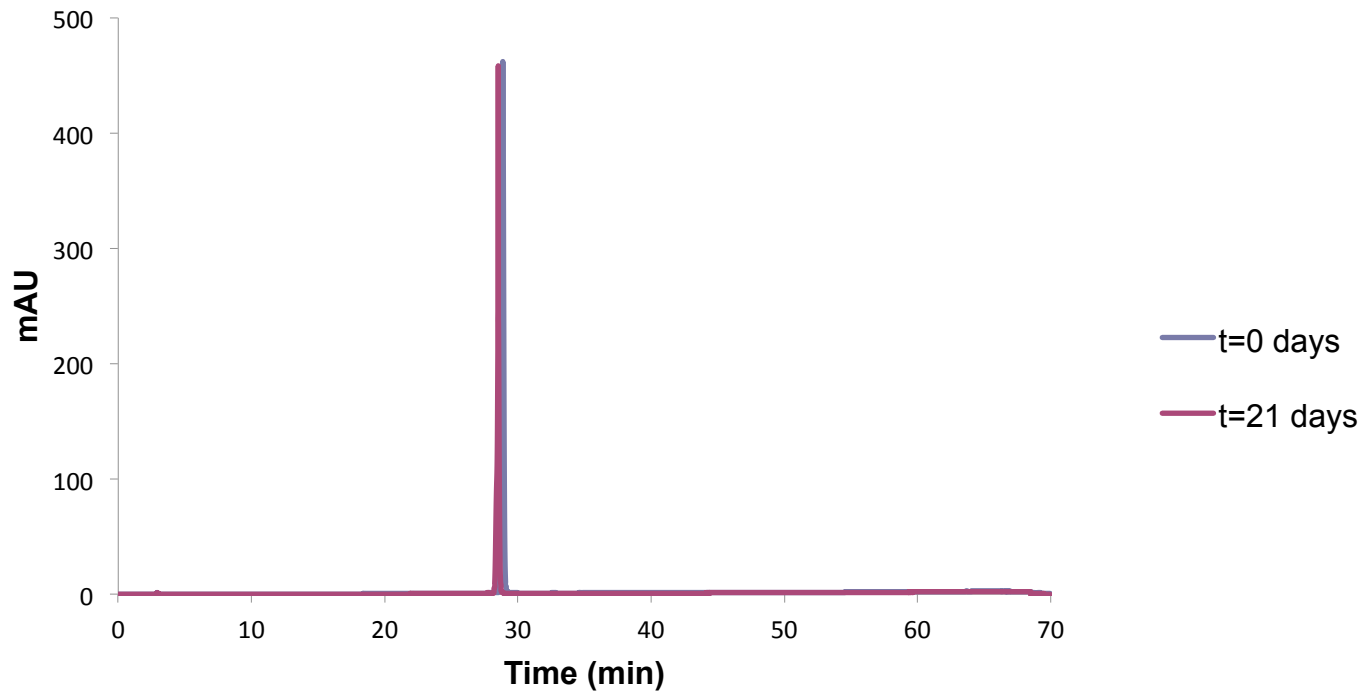

Supplement: Figure S7 — Stability of mt-Cbl conjugate. The conjugate was incubated for three weeks in mouse plasma at 37°C. Its purity was then assessed using HPLC and one peak was noted, suggesting that the conjugate was stable in mouse plasma. (PDF) [file pone.0060253.s007.pdf]

*Stability in Saline*

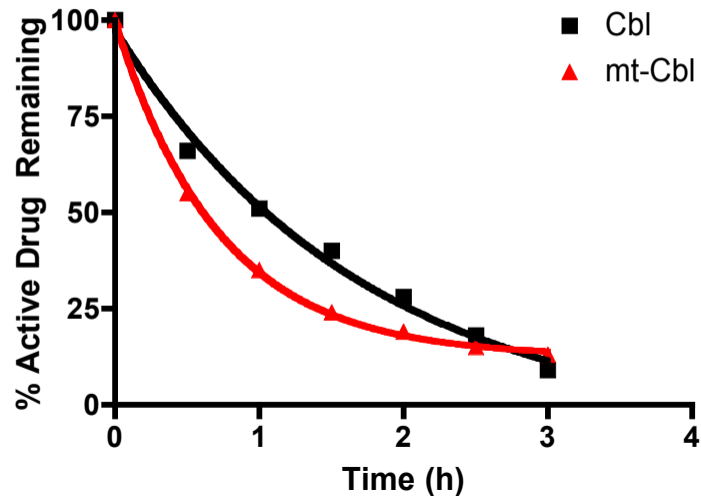

*Stability in Plasma*

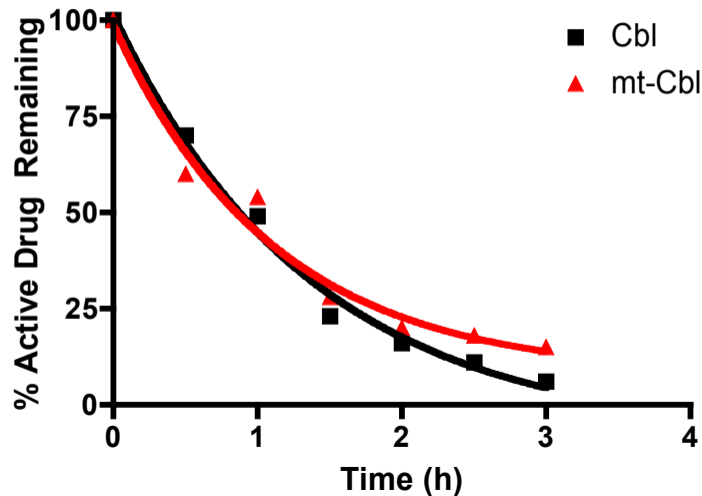

Supplement: Figure S8 — Half-life of mt-Cbl and Cbl. The mt-Cbl and Cbl compounds were incubated for varying times in saline or mouse plasma at 37°C. The percentage of active drug was monitored via HPLC-MS/MS. Both compounds showed comparable T1/2 of 1 h in saline and plasma. (PDF) [file pone.0060253.s008.pdf]

*Unt*

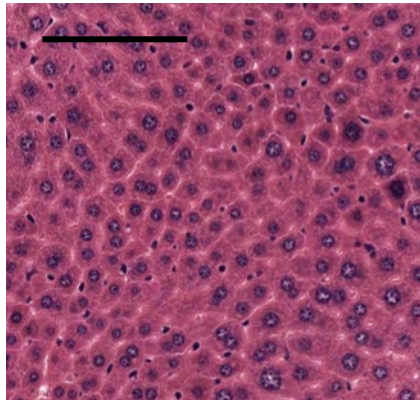

*Cbl*

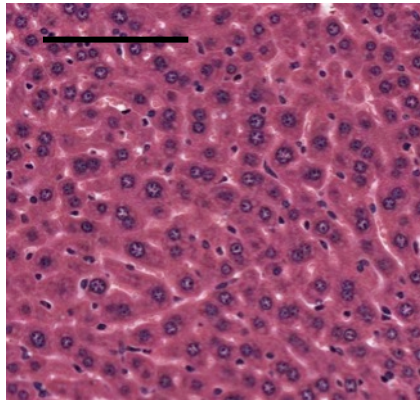

*Mt-Cbl*

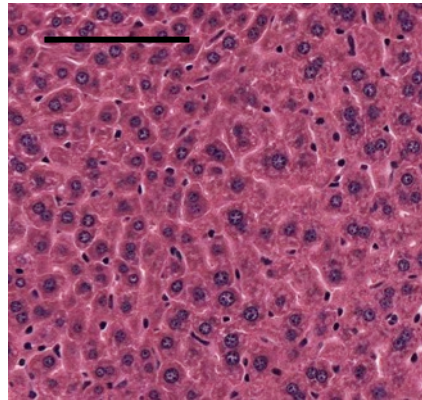

Supplement: Figure S9 — Immunohistochemistry of mouse livers following treatment. H&E staining was performed in a standard manner on livers from untreated mice or those treated with equimolar doses of Cbl (3.7 mg/kg) and mt-Cbl (15 mg/kg). All livers were histologically normal with no features of injury or toxicity. Images were reviewed by a pathologist with liver expertise. Representative images are shown above. Scale bar, 100 μm. (PDF) [file pone.0060253.s009.pdf]
